# Costs and effects of intra-operative fluorescence molecular imaging – A model-based, early assessment

**DOI:** 10.1371/journal.pone.0198137

**Published:** 2018-06-01

**Authors:** Maximilian Präger, Marion Kiechle, Björn Stollenwerk, Christoph Hinzen, Jürgen Glatz, Matthias Vogl, Reiner Leidl

**Affiliations:** 1 Institute of Health Economics and Health Care Management, Helmholtz Zentrum München (GmbH)—German Research Center for Environmental Health, Neuherberg, Germany; 2 Center for Hereditary Breast and Ovarian Cancer, Department of Gynecology, Klinikum Rechts der Isar, Technical University Munich (TUM), Munich, Germany; 3 Comprehensive Cancer Center Munich (CCCM), Munich, Germany; 4 Institute of Biological and Medical Imaging, Helmholtz Zentrum München (GmbH)—German Research Center for Environmental Health, Neuherberg, Germany; 5 Chair for Biological Imaging, Technical University Munich, Munich, Germany; 6 Munich Center of Health Sciences, Ludwig-Maximilians-Universität München, Munich, Germany; Stanford University, UNITED STATES

## Abstract

**Introduction:**

Successful breast conserving cancer surgeries come along with tumor free resection margins and account for cosmetic outcome. Positive margins increase the likelihood of tumor recurrence. Intra-operative fluorescence molecular imaging (IFMI) aims to focus surgery on malignant tissue thus substantially lowering the presence of positive margins as compared with standard techniques of breast conservation (ST). A goal of this paper is to assess the incremental number of surgeries and costs of IFMI vs. ST.

**Methods:**

We developed a decision analytical model and applied it for an early evaluation approach. Given uncertainty we considered that IFMI might reduce the proportion of positive margins found by ST from all to none and this proportion is assumed to be reduced to 10% for the base case. Inputs included data from the literature and a range of effect estimates. For the costs of IFMI, respective cost components were added to those of ST.

**Results:**

The base case reduction lowered number of surgeries (mean [95% confidence interval]) by 0.22 [0.15; 0.30] and changed costs (mean [95% confidence interval]) by €-663 [€-1,584; €50]. A tornado diagram identified the Diagnosis Related Group (DRG) costs, the proportion of positive margins of ST, the staff time saving factor and the duration of frozen section analysis (FSA) as important determinants of this cost.

**Conclusions:**

These early results indicate that IFMI may be more effective than ST and through the reduction of positive margins it is possible to save follow-up surgeries–indicating further health risk–and to save costs through this margin reduction and the avoidance of FSA.

## Introduction

Breast cancer is the most common cause of cancer deaths in women in Germany. 30.8% of all cancer incidence in women in 2012 were caused by the disease [1].

In recent years many innovative technical methods have been developed to detect and treat breast cancer [2–5]. There are some methods applied by the surgeon, e.g. radiofrequency spectroscopy, which can be used to examine the margin status of a tumor during surgery [6]. To assess the margin status the tumor with surrounding tissue is removed. In the case of having malignant cells at the resection edge the classification is called positive margins, otherwise it is called negative margins [7, 8]. A person with positive margins has an elevated risk for breast cancer recurrence [9, 10]. Therefore a common consensus between surgeons is to further resect this type of margins in order to achieve negative margins [11]. Another often used procedure of breast cancer surgery is the removal of the sentinel lymph node. Some techniques use the fluorescent dye indocyanine green (ICG). This dye has a very high detection rate, ranging from 73.1% to 100% depending on the other components of the dye [6].

The type of recurrence also plays an important role in the course of the disease. Local recurrence means that the tumor comes back to the place of origin after some time, whereas regional recurrence indicates that the tumor returns to the lymph nodes near to the origins of the tumor [12]. The worst prognosis is given in the case of metastases. This type of recurrence occurs in the more distant parts of the body, e.g. the brain, the liver, or the bones [12]. Later occurrence of secondary tumors is not considered in this analysis.

Various techniques for breast conserving therapy exist [13]. Beside preoperative techniques of tumor localization especially the assessment of margins plays an important role. An often used strategy of margin assessment is frozen section analysis (FSA). Combined with current, standard techniques of breast conserving surgery (ST) this is chosen as the reference technique in this study [14]. The frozen and dissected tissue is examined by a pathologist and after the diagnosis the surgeon is informed. An advantage of this method is the fact that it can be applied by the surgeon during surgery [15].

Intra-operative fluorescence molecular imaging (IFMI) is an innovative surgical method of breast cancer imaging [16]. It can be used to detect the margin status and sentinel lymph nodes during surgery. In order to make the tumor visible for the surgeon, a fluorescence molecular agent, for example Bevacizumab-IRDye800CW containing the monoclonal antibody Bevacizumab targeting the vascular endothelial growth factor A, is injected into the patient. The optical imaging system usually consists of a fluorescence and a white light camera and the resulting images can be examined on screens at the operating room [17]. A phase I study in which IFMI was used took place in the Netherlands; some data from this trial is used to inform our model parameters [18]. Within this phase I study, besides patient-safety as the primary endpoint, tumor and tumor-margin uptake of Bevacizumab-IRDye800CW could be confirmed [19]. In image-validation, a sufficient labelling performance was demonstrated [20]. Therefore, compared to ST, IFMI is expected to reduce the number of surgeries and the costs as a consequence of the avoided surgeries and the avoidance of FSA.

The objective of the study is to analyze short term effects of IFMI compared to ST by reducing the presence of positive margins after surgery. The effects considered here include the avoided number of surgeries and the cost savings measured in incremental costs. Developing and using a decision tree model effects could be calculated such that the study aim was reached.

## Methods

### Model structure

**Fig 1:**
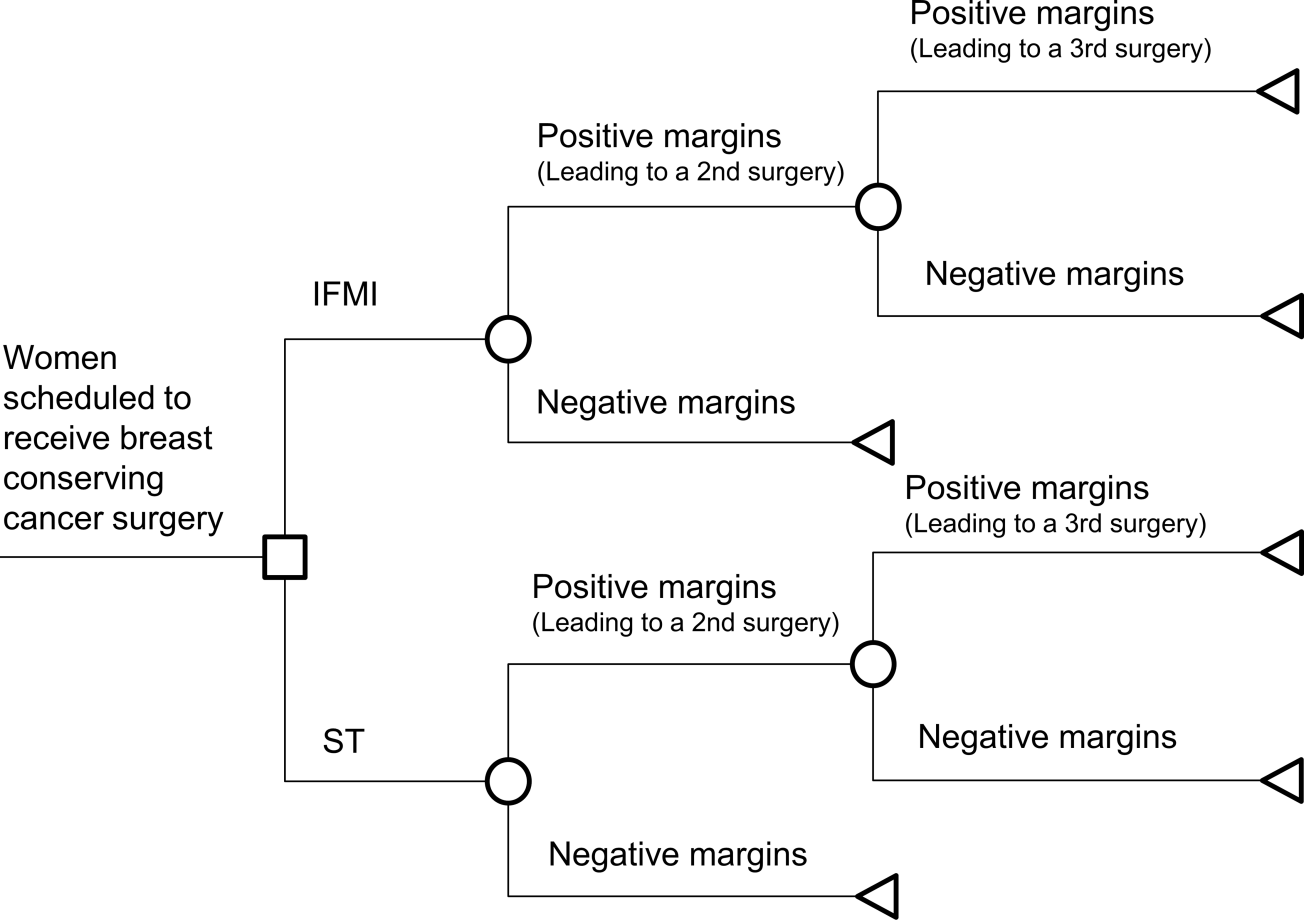
Structure of the decision tree. IFMI = intra-operative fluorescence molecular imaging. ST = standard techniques of breast conserving surgery.

Decision trees are a basic type of decision-analytic models, which is commonly used to assess the short term consequences of interventions [21]. To assess the costs and consequences of IFMI and ST, we developed a decision tree, which is illustrated in Fig 1. When designing this decision model, we followed the good modelling practice guidelines, as published by Philips et al. 2006 [22]. Both the IFMI and the ST strategies were implemented in the model’s tree structure (Fig 1): Within the model structure it is accounted for the situation in which a surgery has been completely finished and the pathological report indicates the probabilities of occurrence of the two margin types [23]. IFMI is applied within the first surgery whereas for the following surgeries probabilities of the margins are assumed the same both for the IFMI and the ST path. Due to the consensus that positive margins should be removed in most cases, we assume a follow-up surgery in case of positive margins, whereas in case of negative margins no further breast cancer surgery takes place [8, 24]. A third surgery is assumed to be the final surgery if both the first and the second surgery yielded positive margins (see Fig 1).

The time horizon considered within analysis is the time between the first breast cancer surgery and return to work after the last surgery needed to finally achieve negative margins.

### Costs

Surgical costs are calculated from a hospital perspective. In addition, we accounted for loss of productivity. The costs needed for calculations were mainly costs for the standard technique, costs of the devices for surgery, staff costs, costs of the fluorescent agent Bevacizumab-IRDye800CW, savings due to the avoidance of FSA, costs regarding the prolongation of surgery due to the application of IFMI and lost productivity costs. Table 1 gives an overview of main cost parameters used in the model. For the costs of a certain model path the respective cost components are added up.

**Table 1:** Parameters related to costs per surgery.

Cost category [unit]	Base case	Distribution for probabilistic analysis	Tornado analysis	Further sensitivity analyses	Sources
Proportion of positive margins after first surgery with IFMI	0.1	Beta (SE = 0.018)	0.075; 0.125	Relative Risks (range 0–1) multiplied with ST reference value 0.3	[18, 32], med. experts
Proportion of positive margins after first surgery with ST	0.3	Beta (SE = 0.051)	0.225; 0.375	0.183(SE = 0.035)	[32–35]
Costs of a breast cancer surgery with current standard techniques [€]	3,508	Gamma (SE = 175)	2,631; 4,385	2,201(SE = 110); 5,047(SE = 252)	[32, 36]
Costs of change in the duration of surgery due to IFMI, input for calculation					
Duration of a standard breast cancer surgery [minutes]	59	Triangular (min = 35, max = 83)	44.25; 73.75	35(min = 11,max = 59); 83(min = 59,max = 107)	[32, 37]
Prolongation due to IFMI: [minutes]	10	Triangular (min = 5, max = 15)	7.5; 12.5	-	[18, 32]
Duration of frozen section analysis [minutes]	27	Triangular (min = 13, max = 53	20.25; 33.75	13(min = 0,max = 26); 53(min = 40,max = 66)	[32, 38]
Staff time saving factor [no dimension]	0.64	-	0.48; 0.8	0; 1	Calculation based on med. experts, [32]
Cost of additional staff for IFMI [€]	107	Gamma (SE = 5)	80; 134	-	[32, 36]
Cost per case, materials [€]					
Bevacizumab-IRDye800CW	500	Gamma (SE = 25)	375; 625	800(SE = 40)	[18, 32, 39]
Camera system	182	Gamma (SE = 18)	137; 228	-	[18, 32]
Sterile draping	23	Gamma (SE = 2)	18; 29	-	[32, 40]
Lost productivity per case [€]	521	Gamma (SE = 52)	390; 651	-	[32, 41, 42]

SE = standard error, min = minimum value, max = maximum value, med. = medical, IFMI = intra-operative fluorescence molecular imaging, ST = standard techniques of breast conserving surgery.

The costs of ST were derived as a lump sum from the German Diagnosis Related Group (DRG) system. DRGs relevant for ST were identified using the German version of the International Classification of Procedures in Medicine (ICPM) which is called “Operationen- und Prozedurenschlüssel” (OPS). The DRGs then were weighted and combined according to the frequency of occurrence among the breast conserving OPS procedure which leads to a weighted average cost as well as an underlying averaged two dimensional matrix combining cost centers and cost categories [25]. These costs are multiplied with numbers of surgeries of a given model path as this cost component appears in each surgery.

To account for IFMI the additional costs needed as compared to ST were calculated. As IFMI was used for the first surgery only the respective costs are added once for the IFMI path. Additional staff costs of IFMI were derived by multiplying the staff costs within the mentioned matrix for ST by factors reflecting the additional staff need of IFMI. Additional staff is assumed to be present during the whole surgical procedure.

The IFMI device was recognized with total costs of €150,000 according to the trial data. Additionally, maintenance costs of 10% p.a. of the original price of the device were used. In order to determine costs of the device per surgery, the operational life span of the device was assumed to be 7 years according to standard life spans of video systems [26]. Furthermore, 200 breast conserving surgeries per year of a midsize women’s hospital were used for relating equipment cost to surgeries [27, 28].

The application of IFMI additionally requires 10 minutes for fluorescence inspection during surgery. Furthermore, a shortening of surgical time takes places by avoiding waiting times for the results of FSA. To adjust for the fact that only parts of the medical staff have to stay with the patient a staff time saving factor (range: 0–1) is multiplied with the duration of FSA. The factor indicates the proportion of time of FSA which can be saved. Based on interviews of two surgeons it is assumed that the senior physician’s time cannot be saved; accounting for German wage structure this renders a staff time saving factor of 0.64 which is taken for the base case. The difference between the prolongation and the shortening is then multiplied with the costs per minute of surgery which is derived by dividing the weighted average matrix mentioned above by the expected duration of a breast conserving surgical procedure.

Taking into account productivity losses of patients, indirect costs were also calculated. If an additional surgery is needed because of the presence of positive margins the patient has to stay additional time in hospital and in rehabilitation before she can return to work. For indirect costs, average wage per day is multiplied by working days lost per surgery, the proportion of women in employment in German general population, and the quantity of surgeries of the corresponding model path. The working days lost between two surgeries and between the last surgery and the final return to work are assumed to be 14 days each [29, 30].

An overview on the combination of cost components in each path of the model is given in Table 2. All costs were converted in Euros where necessary using purchasing power parity adjusted exchange rates regarding the gross domestic product [31].

**Table 2:** Cost components linked to the model paths in the base case.

Path	Cost Components
Positive margins after the first surgery, application of IFMI 1) Positive margins after the second surgery (i.e. three surgeries) 2) Negative margins after the second surgery (i.e. two surgeries)	• Costs of a breast cancer surgery 1): three times, 2): twice ^a^ • Additional costs of an application of IFMI (once) ^a^ • Lost productivity (1: three times, 2: twice)
Negative margins after the first surgery, application of IFMI (i.e. one surgery)	• Costs of a breast cancer surgery (once) ^a^ • Additional costs of an application of IFMI (once) ^a^ • Lost productivity (once)
Positive margins after the first surgery, application of ST 1) Positive margins after the second surgery (i.e. three surgeries) 2) Negative margins after the second surgery (i.e. two surgeries)	• Costs of a breast cancer surgery 1): three times, 2): twice • Lost productivity (1: three times, 2: twice)
Negative margins after the first surgery, application of ST (i.e. one surgery)	• Costs of a breast cancer surgery (once) • Lost productivity (once)

IFMI = Intra-operative fluorescence molecular imaging

ST = standard techniques of breast conserving surgery.

^a^ Costs of breast cancer surgery and additional costs of an application of IFMI can be summarized as costs per IFMI-surgery. The additional costs consist of the device, Bevacizumab and the dye, costs due to prolongation of operation time, savings due to the avoidance of FSA, costs of a sterile draping and costs regarding additional staff

### Proportion of positive margins and relative risk assigned to the tree structure

The probability of having positive margins after ST as first surgery was derived from the literature; this proportion of positive margins currently ranges between 20% and 40% [33, 34]. We therefore implemented a baseline point estimate of 30% positive margins for ST, and assumed a standard error of 0.051. After considering trial documentation and consultation of medical experts, we assumed 10% positive margins after the first surgery with IFMI as the base case [18]. This reduction by IFMI can be expressed in terms of relative risk, equaling 33.3% for the base case. As no strong evidence is available we performed sensitivity analyses covering the whole range of possible reductions from 0% to 30% positive margins left after the first surgery using IFMI. Some of the cases scheduled for a second surgery need a third surgical procedure because of the presence of positive margins. Given that in the literature estimates of a third surgery, i.e. the proportion of positive margins after the second surgery, range between 6% and 13%, we implemented a point estimate of 10% and a standard error of 0.018 [23, 43–45]. Standard errors were calculated based on the Gaussian distribution, assuming uncertainty ranges corresponding to 95% confidence intervals. The proportion of third surgeries is both applied to the ST and IFMI paths.

### Base case scenario

Endpoints were the amount of surgeries saved and incremental costs. The incremental number of surgeries reflects the difference in number of surgeries expected in IFMI and in ST. Using the corresponding costs and analogous calculation, expected costs were derived for each treatment path and incremental costs again calculated as the difference between the two paths.

### Sensitivity analysis

The effectiveness of using IFMI as first surgery remains to be determined. We present model results for this strategy achieving positive margins levels of 0%, 5%, 10%, 15%, 20%, 25% and 30%, corresponding to a relative risk of 0, 0.17, 0.33, 0.5, 0.67, 0.83 and 1. Both point estimates and 95% confidence intervals were linearly interpolated to derive continuous estimates. This approach is supported by the linear character of the model structure. Point estimators could be derived exactly by this method whereas confidence intervals could be derived approximately. Within one graph all other variables besides the relative risk were held constant.

For the probabilistic analysis, gamma distributions were assigned to the costs, whereas a triangular distribution was used for the duration of ST, the prolongation time due to IFMI and the shortening of time by avoiding FSA. For the cost parameters the standard error was assumed to be 10% of the point estimator if values were more uncertain, e.g. if some critical assumptions were made. Otherwise the standard error was set to 5% of the point estimator. For the construction of the confidence intervals 10,000 draws from the distributions were performed within Monte Carlo Simulation.

Deterministic sensitivity analyses are shown in similar graphs including confidence intervals. A tornado diagram shows the ranking of relative influence of individual variables on results. The high and the low value used to set up the tornado diagram were calculated for each variable using the increment and the decrement of 25 percent of the mean value [32]. Across the potential range of effectiveness of IFMI, the impact of the most influential variables is then tested in further sensitivity analyses.

An upper limit of DRGs for sensitivity analysis could be identified from literature. The case is described with a main diagnosis of breast cancer and the other diagnoses were non-insulin-dependent diabetes mellitus with unspecified complications, dilated hypertrophic cardiomyopathy and sequelae of cerebral infarction. Further details can be taken form the source [46]. Using the two OPS codes of breast conserving surgery and lymphadenectomy this leads to a DRG of €5,047. The lower limit could not be determined by literature such that the lowest DRG used within the calculations of the average matrix was taken.

During ST the surgeon and the other team members have to wait for the results of pathologic examination of FSA. For the base case a staff time saving factor was applied to the savings of FSA reflecting the fact that not the whole staff has to stay with the patient during waiting time. Within another sensitivity analysis this factor is set to 1 in order to provide a scenario in which the whole time of FSA can be saved.

Evidence suggests that 59 minutes per surgery could be seen as an expected duration of ST. If breast reconstruction is integrated into the breast conserving operation time increases to 83 minutes [37]. Therefore we extend the duration of ST to 83 minutes in a further sensitivity analysis and we also used the duration of 35 minutes within another analysis to account for a shorter operation time.

Input data for the model were taken from a phase I trial completed in 2014. In order to test alternative scenarios of recent clinical practice, both sensitivity analysis concerning a higher cost of Bevacizumab-IRDye800CW and a lower share of positive margins found within ST were performed. For Bevacizumab-IRDye800CW a high cost level for contrast agents was tested [39]. Furthermore, recent findings for positive margins of ST were integrated into the analysis [35].

**Fig 2:**
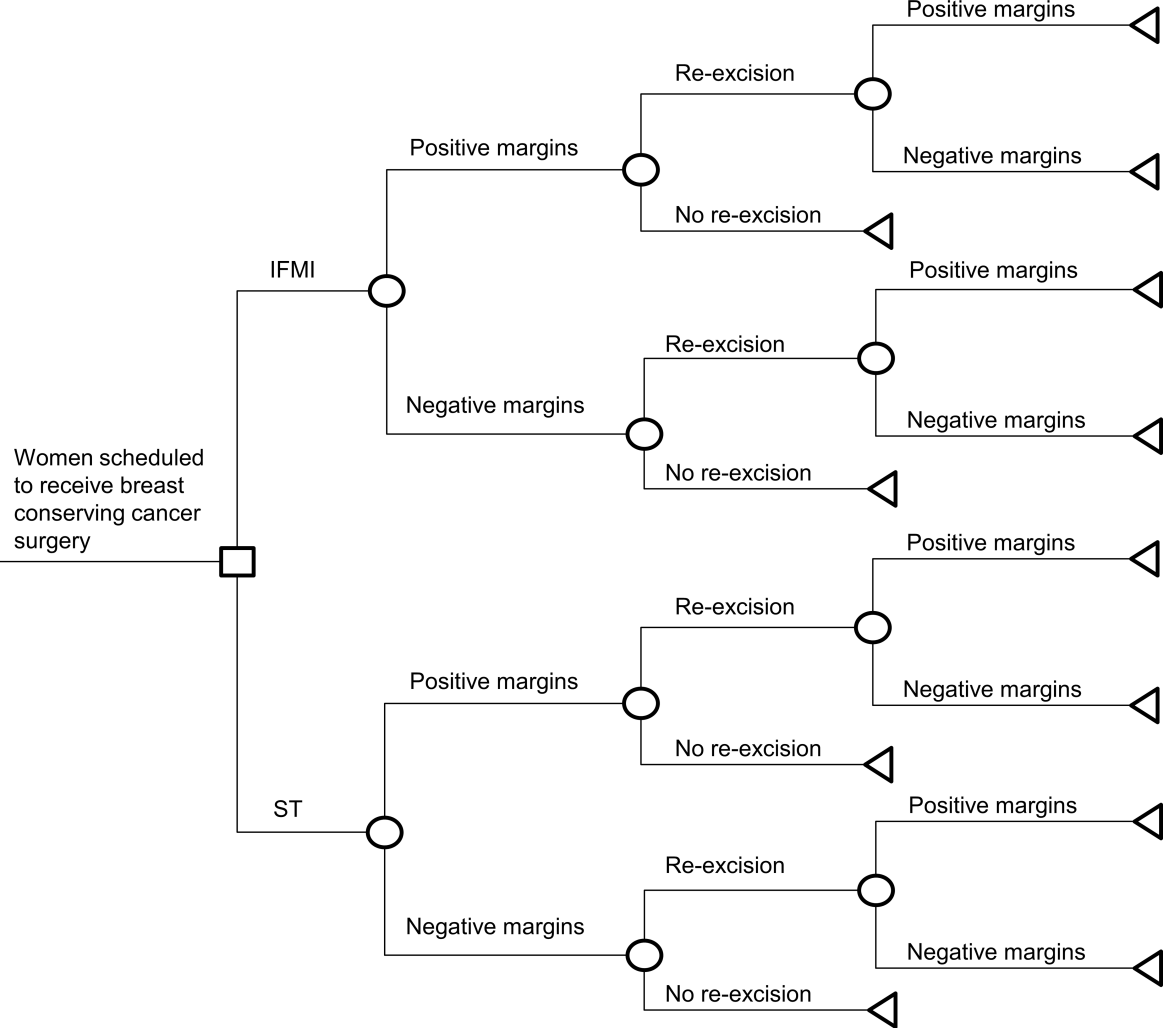
Structural sensitivity analysis: Inclusion of no re-excision of positive margins, excision of negative margins. IFMI = intra-operative fluorescence molecular imaging. ST = standard techniques of breast conserving surgery.

**Fig 3:**
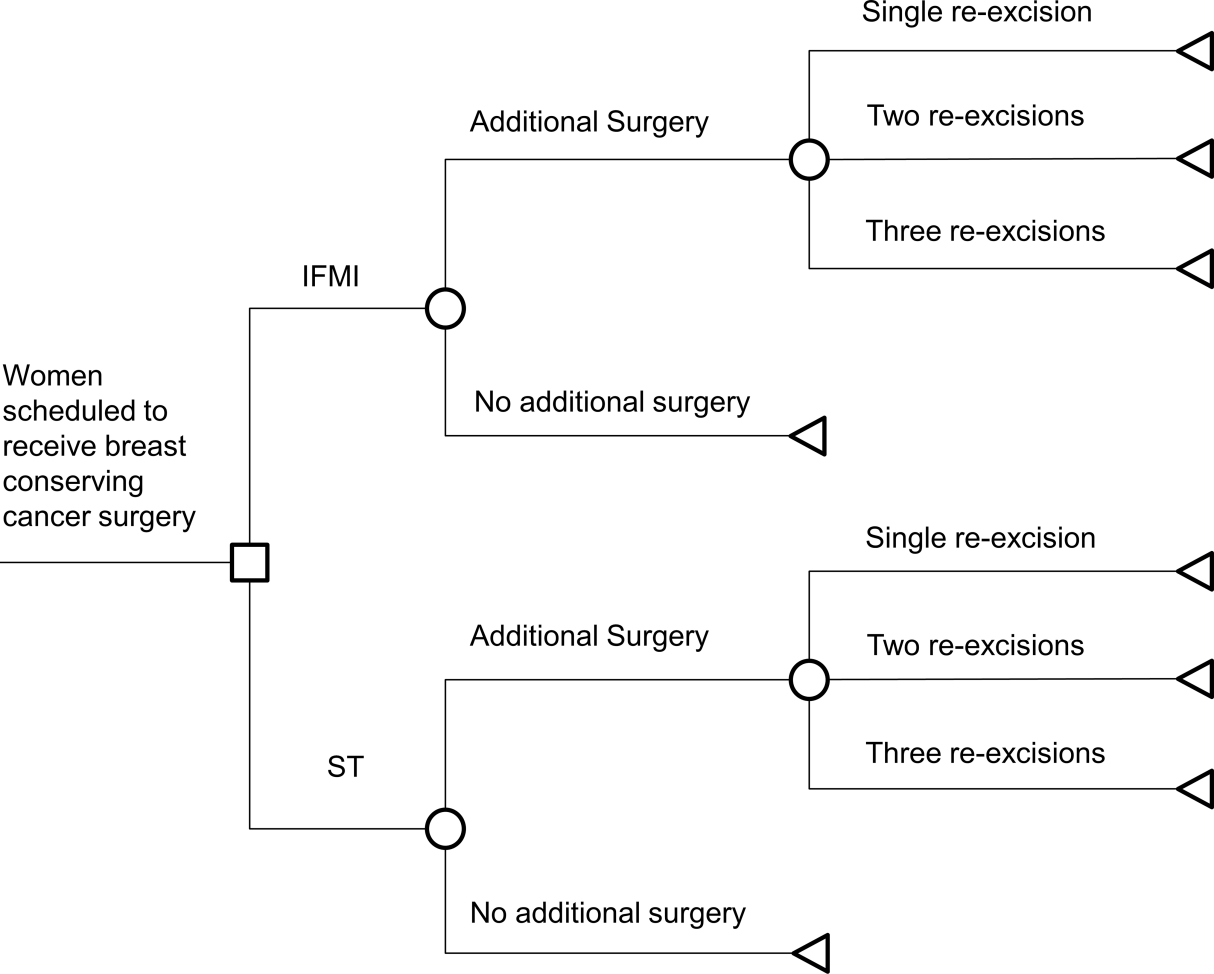
Structural sensitivity analysis: Numbers of surgeries without margin dependency. IFMI = intra-operative fluorescence molecular imaging. ST = standard techniques of breast conserving surgery.

According to McCahill et al. less than 100% of persons with positive margins are re-excised and also some people with negative margins are operated again [11]. In a structural sensitivity analysis we thus considered that both patients with positive and with negative margins have a positive probability of being re-excised or not being re-excised after the first surgery (Fig 2). For the following surgeries every person with positive margins is assumed to be re-excised, whereas each person with negative margins is assumed not to be re-excised. Probabilities of third surgeries were assumed to stay the same. In another analysis, using again data of McCahill et al., we explored the effect of fourth surgeries in which the actual proportions of numbers of breast conserving cancer surgeries without stratification by margin type are given (Fig 3).

### Software

The cost matrix of a breast conserving surgery according to the German DRG-system is derived from G-DRG-Report-Browser 2017 [36]. In order to find specific DRGs for sensitivity analysis the DRG web grouper of the university hospital of Münster was used [47]. The model was set up and analyzed using TreeAge Pro 2012 [48]. Some calculations and generating of figures was done using the statistical software R version 3.3.2 [49]. The structure of the model and the structural sensitivity analyses were drawn using Microsoft PowerPoint 2010.

## Results

**Fig 4:**
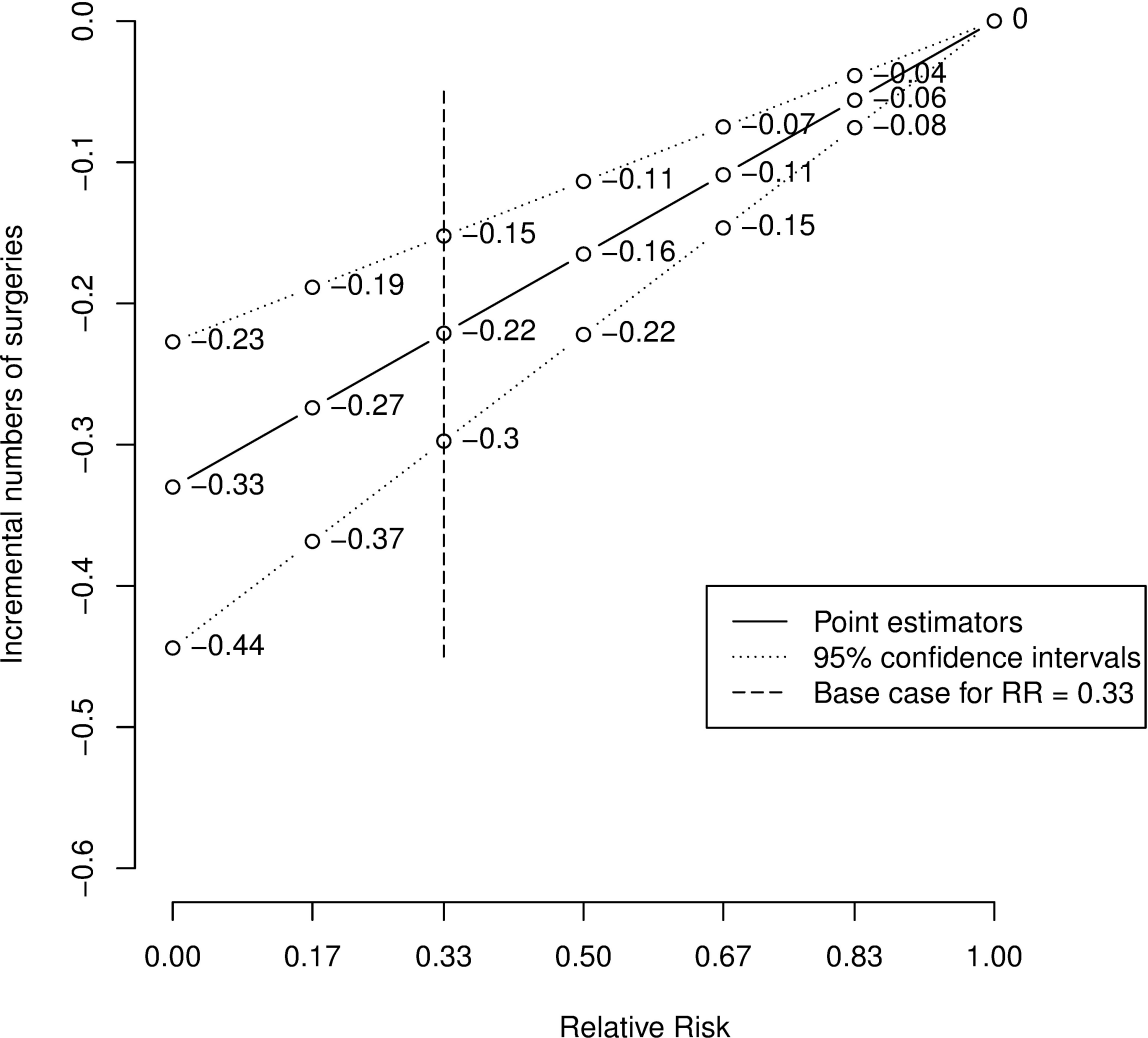
Base case graph: Incremental numbers of surgeries of IFMI vs. ST. RR = Relative Risk.

**Fig 5:**
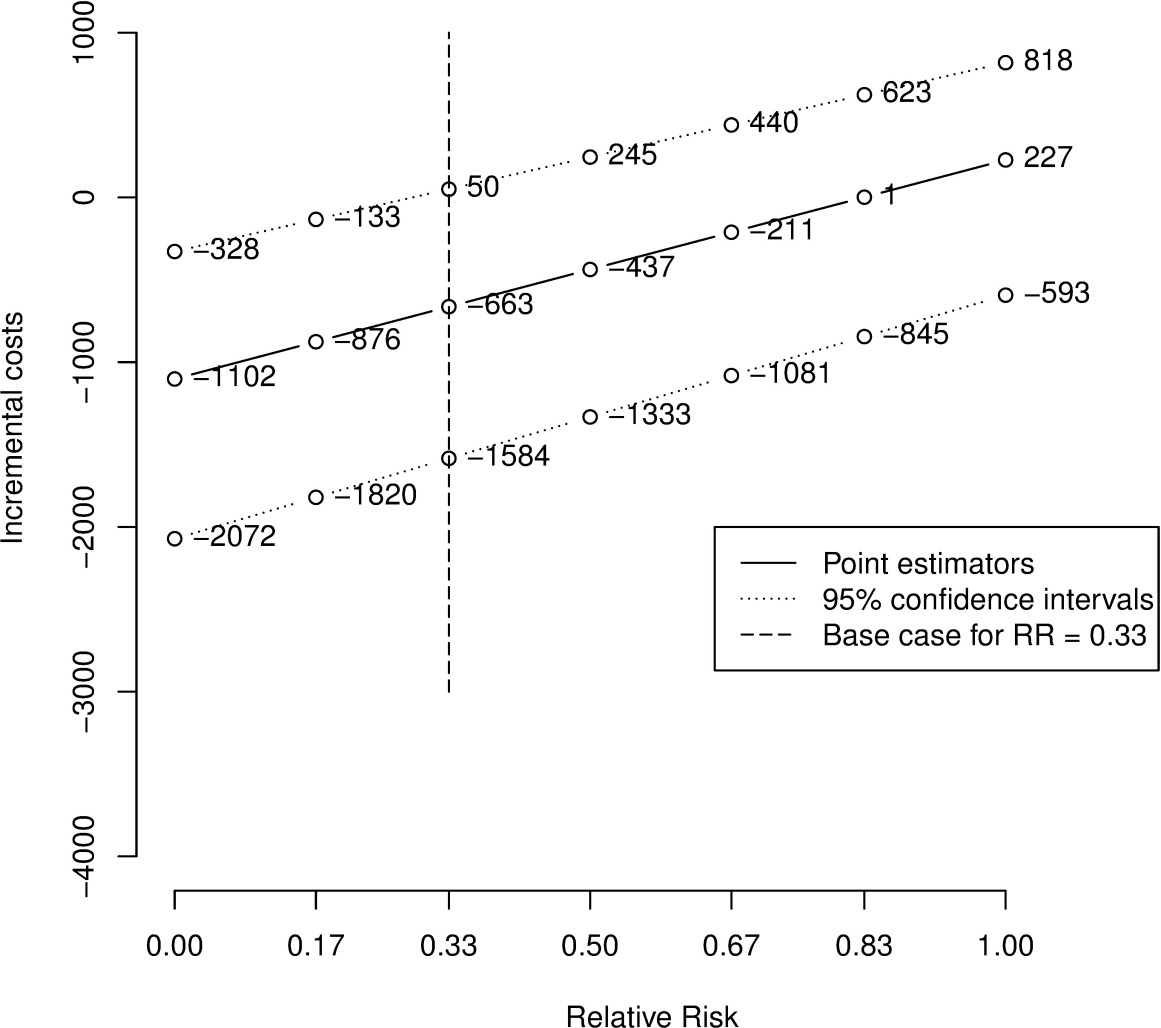
Base case graph: Incremental costs of IFMI vs. ST. RR = Relative Risk.

Applying the base case relative risk of 0.33 the amount of expected surgeries per person using IFMI is 1.11. The ST strategy results in an expected number of surgeries of 1.33. Therefore the incremental number of surgeries (mean [95% confidence interval]) is -0.22 [-0.30; -0.15]. The corresponding results regarding the costs are €4,695 for IFMI and €5,358 for ST, resulting in incremental costs of €-663 [€-1,584; €50] by linear interpolation. Results of the whole spectrum of relative risks calculated by linear interpolation are shown in Figs 4 and 5 in which the base case is marked by a vertical bar.

**Fig 6:**
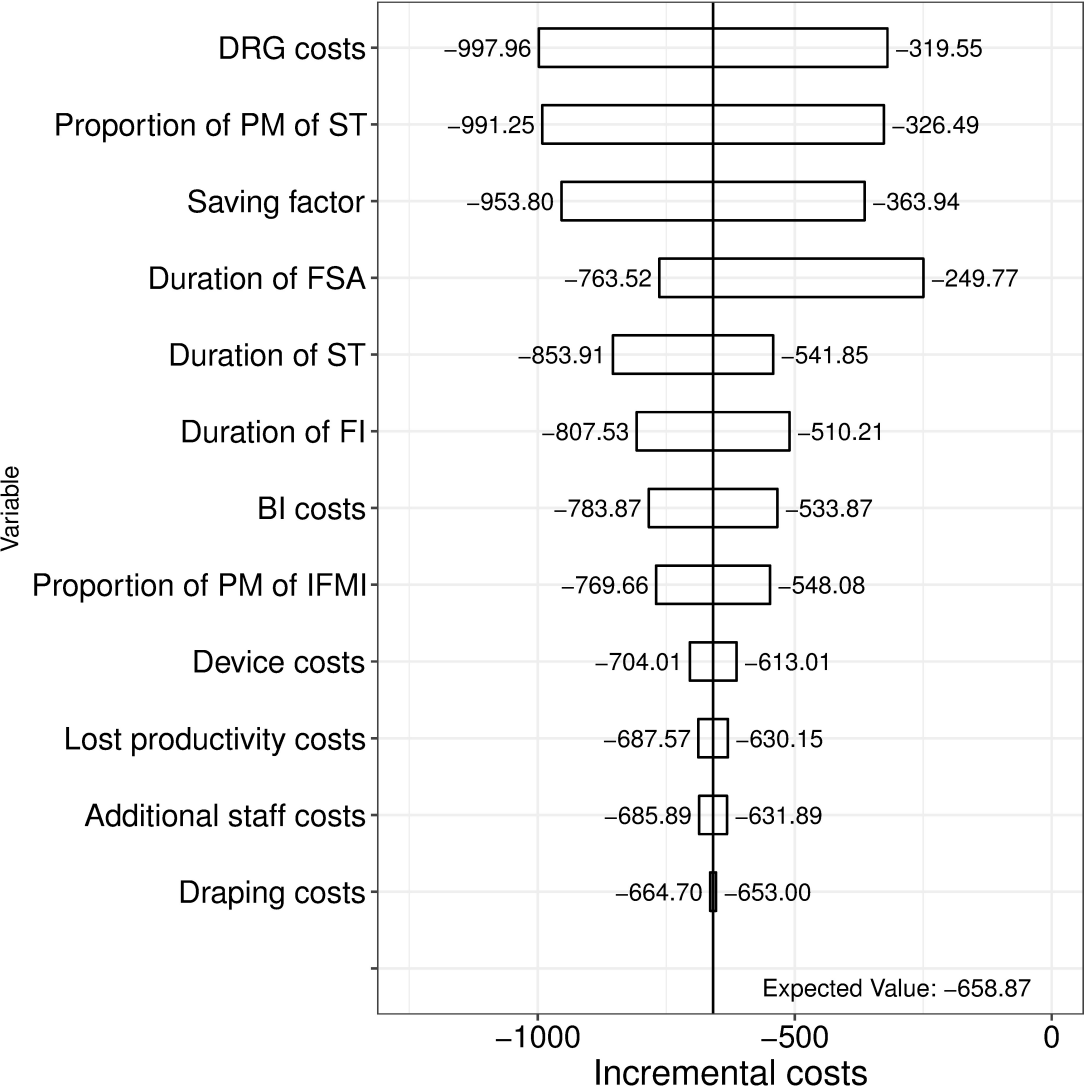
Tornado analysis: Incremental costs of IFMI vs. ST. DRG = Diagnosis Related Group, PM = positive margins. ST = standard techniques of breast conserving surgery, FSA = frozen section analysis. FI = fluorescence inspection, BI = Bevacizumab-IDRye800CW.

The most important cost drivers of the intervention are shown in the tornado diagram (Fig 6). Besides the probability of having a certain margin type especially the DRG costs, the staff time saving factor, the duration of FSA and the duration of ST play an important role.

Regarding sensitivity analyses compared to the base case, increasing the DRG costs leads to a downward shift of the incremental costs, the slope becomes steeper and uncertainty increases. The opposite direction of the effects can be seen when the DRG costs are decreased (Fig A in S1 Fig and Fig B in S1 Fig).

Furthermore, setting the staff time saving factor for waiting times of FSA to unity leads to a downwards shift of the incremental costs while uncertainty increases (Fig C in S1 Fig)–on the other hand, assuming no staff time could be saved at all would render incremental costs of €516 [€94; €1,000] for a relative risk of 0.33. The same result also would appear if the surgeon orders FSA after an application of IFMI in order to get additional validation regarding margin results. If the duration of FSA is raised within analysis the incremental costs are reduced for all relative risks while lowering the duration of FSA results in an upwards shift together with a reduction of uncertainty (Fig D in S1 Fig and Fig E in S1 Fig). Increasing the duration of ST results in an upward shift of the incremental costs together with a reduction of uncertainty, whereas decreasing the duration of ST results in the opposite effect (Fig F in S1 Fig and Fig G in S1 Fig). Within all sensitivity analyses described above a shift downwards of the incremental costs features a linear influence of these variables on model results, and the costliness of IFMI compared to ST improves independent of relative risks, whereas a shift upwards worsens it, respectively.

Higher costs of Bevacizumab-IRDye800CW of €800 lead to an upward shift of the incremental costs and the confidence intervals (Fig H in S1 Fig). In the case of a lower proportion of positive margins within ST the slope of the incremental costs and the uncertainty predominantly decreases which results in a worsening of the costliness of IFMI, especially for the lower relative risks (Fig I in S1 Fig).

The first case of structural sensitivity analysis describes the situation in which both re-excision of negative margins and no re-excision of positive margins are possible. In the second case further surgeries do not depend on the type of margins after the surgery. The cost scenario of the first case worsens the costliness of IFMI vs ST while the cost scenario of the second case improves it (Fig J in S1 Fig and Fig L in S1 Fig). In the first structural sensitivity scenario, the numbers of surgeries saved are also reduced respectively (Fig K in S1 Fig). Incremental numbers of surgeries of the second case are not shown here as the results were nearly the same as in the base case graph.

## Discussion

In our base case IFMI saves 0.22 surgeries per person scheduled to receive breast conserving therapy. The more the proportion of positive margins was reduced by IFMI the more surgeries could be avoided. While future trials will show stronger evidence regarding the effect of IFMI, we developed a model framework to analyze possible results at a very early stage. Results of a phase I study were used as a base case, rendering a first possible order of magnitude of the effects of IFMI on number of surgeries and costs. In order to address uncertainty, the whole range of possible margin reductions was investigated. By considering up to three operations per person to finally achieve negative margins the model also covers a wide range. For more detail, sensitivity analyses revealed the most important determinants of results, for example, the DRG costs. These influential variables indicate need for future consideration both in patient management as well as in data collection, for more accurate analysis. In structural sensitivity analysis it was shown that consideration of re-excisions for negative margins and no re-excisions for positive margins reduced incremental surgeries by about a quarter as compared to the base case.

One key result, the incremental costs of IFMI vs. ST are negative for the base case, i.e. the IFMI intervention is less expensive than the strategy without IFMI, but significant only to a slightly higher level than 5%. Within the intervention, the DRG costs, the proportion of positive margins of ST, the staff time saving factor and the duration of FSA have the highest cost impact. Most of the sensitivity analyses showed significant negative incremental costs for relative risks below 0.33. Furthermore, higher costs of the molecular agent and a lower proportion of positive margins within ST were tested in a sensitivity analysis. The change in the slope for the latter indicated that the potential impact of a reduction in the share of positive margins through the application of IFMI has diminished.

In the model, costs of IFMI have been assumed using data of a clinical trial. If IFMI will be applied within a daily clinical practice, costs would most likely be reduced through the higher rate of breast cancer surgeries. It is likely that e.g. costs of the contrast agent could be reduced as higher volume can be ordered from pharmaceutical companies.

To reflect the additional costs of IFMI versus ST, some additions to the DRGs have been implemented in the model. Financing IFMI for daily usage in hospitals in Germany would thus most likely require a submission to the New Methods of Diagnosis and Treatment (“Neue Untersuchungs- und Behandlungsmethoden” or NUB) procedure. By this procedure, hospitals can negotiate extra reimbursement for new technologies of which the costs would reach beyond the current level of DRG reimbursement [50, 51]. According to the results presented, this would seem to be the case for IFMI.

To improve quality we referred to the checklist of Philips et al. [22]. The structure of our model was checked by medical experts. Data for IFMI was taken directly from a team which is involved in the application of IFMI within a phase I trial in the Netherlands whereas costs of ST were derived from the DRG system. Sensitivity analyses were used to check model logic and results’ consistency.

Because of short term effects being most relevant a decision tree structure seemed adequate. Focusing on the surgical event, integration of the natural course of breast cancer by using a Markov-model did not seem helpful. Furthermore, the linear character of the results made it possible to construct a graph for the whole spectrum of relative risks, thus allowing for interpolation and a flexible focus of the reader on areas of results considered to be relevant.

Some limitations regarding our study exist. The setting is restricted to the German context, e.g. costs of breast cancer cases are taken from the German DRG system. A direct transfer to other countries is not recommended without close consideration of the cost assumptions though the model easily allows for parameter adaptation to other contexts [52]. Within the German DRG system repeated surgeries for the same reason can lead to different types of coding, e.g. combination of the DRGs into a new single DRG [53]. As no system wide information is available regarding the distribution of coding approaches we assume that for each surgery the average DRG is added to the costs of a model path. The calculation for the determination of a specific DRG within breast conserving surgery already includes the cases for two or more surgeries. But as this DRG is reimbursed even for the single surgery cases and the same costs would appear for a hospital for all the following surgeries we multiplied the DRG with the numbers of surgeries for overall costs.

Another restriction is that our analysis has focused on cost consequences and on number of surgeries while the impact on quality of life and thus quality-adjusted life time could not reasonably be included at this early stage.

Beyond, there are more possible consequences of IFMI which are difficult to quantify. For example, reducing surgery may increase availability of time slots in operating rooms and reduce waiting times. Or, patients who can avoid multiple operations might even enjoy better prognosis due to earlier treatment while this would require evidence from future studies. Effects on final positive margins would be another issue which is difficult to address due to the lack of evidence regarding IFMI. Furthermore, false positive readings of IFMI can lead to the excision of healthy tissue or adverse reactions to the contrast agent might occur. Another complicated modeling strategy would be considering hospitals in rural areas, in which surgical efficiency is less compared to hospitals of urban areas. Another limitation, this study could not consider whether cases exist where applying the IFMI-technology could lead to more tissue removed than needed.

Cost effectiveness strongly depends on staff time which can be saved by IFMI. Taking the base case relative risk of 0.33, IFMI would begin to save costs significantly, if about 2/3 of costs of surgery staff for FSA would be saved; the exact value was found between 0.66 and 0.68 depending on run of the probabilistic model. Otherwise, it would be more difficult or even impossible to save costs. For the base case a conservative assumption has been made, however, an accurate estimate would require an own representative survey of the workflow during breast surgery.

Bevacizumab-IRDye800CW plays an important role within the surgical costs of IFMI. This drug can be applied for other cancer types, and optical imaging is not restricted solely to breast cancer [54, 55]. Being able to use IFMI for a broader range of diseases might also lead to cost reductions due to economic effects such as learning curves–reducing time for IFMI application–and economies of scope. Additionally, patent expiration of Bevacizumab is expected in the United States for 2018 [56], and this is most likely to contribute to price reduction over time.

Another area of future application of IFMI is that it seems essential in a surgical field in which re-operations are not possible or very difficult. This is especially the case for patient groups who incur a high risk of complications or even mortality when undergoing surgery [57].

The aim of IFMI is to improve quality of life as a consequence of avoided surgeries. In this early-stage analysis, we were able to indicate ranges for the amount of surgeries saved, and the cost impacts linked to that. The model quantifies the reduction of number of surgeries for patients, an importantly beneficial effect, depending upon the reduction of the share in positive margins. Results also indicate that IFMI might lead to cost savings, especially if waiting times for the results of frozen section analysis can be saved. Key cost drivers were identified of which reduction can be considered in the further development of IFMI strategies.

## Supporting information

S1 Fig Further sensitivity analyses. (DOC)
