# Supplementary material for: Costs and effects of intra-operative fluorescence molecular imaging – A model-based, early assessment
Source: PLoS One. 2018 Jun 1;13(6):e0198137. doi: 10.1371/journal.pone.0198137 (PMC5983425; doi:10.1371/journal.pone.0198137)
Supplement: S1 Fig — (DOC) [file pone.0198137.s001.doc]

**Supporting information:**

**S1 Fig. Further sensitivity analyses**


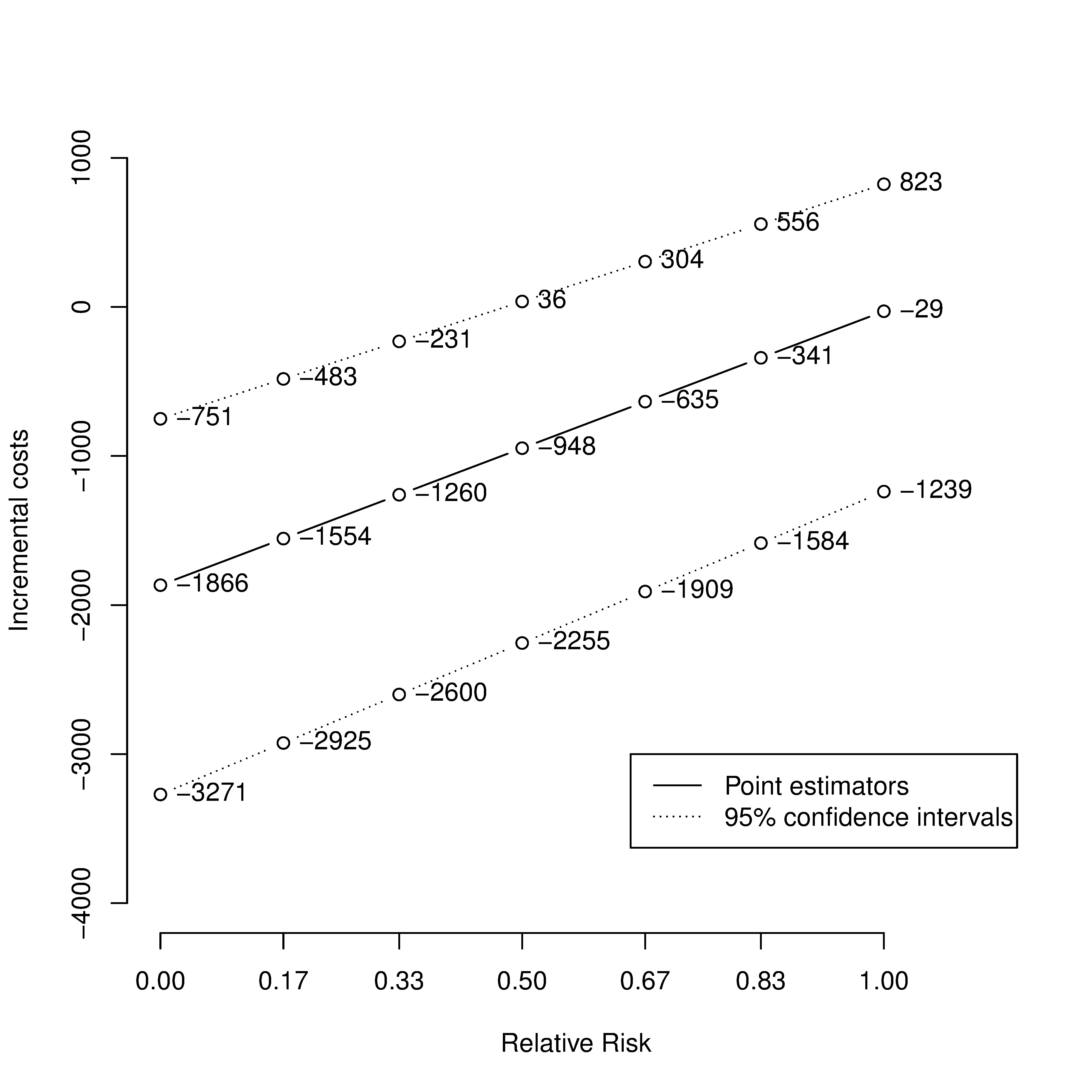


**Fig A. Sensitivity analysis: €5,047 for the DRG.**

**
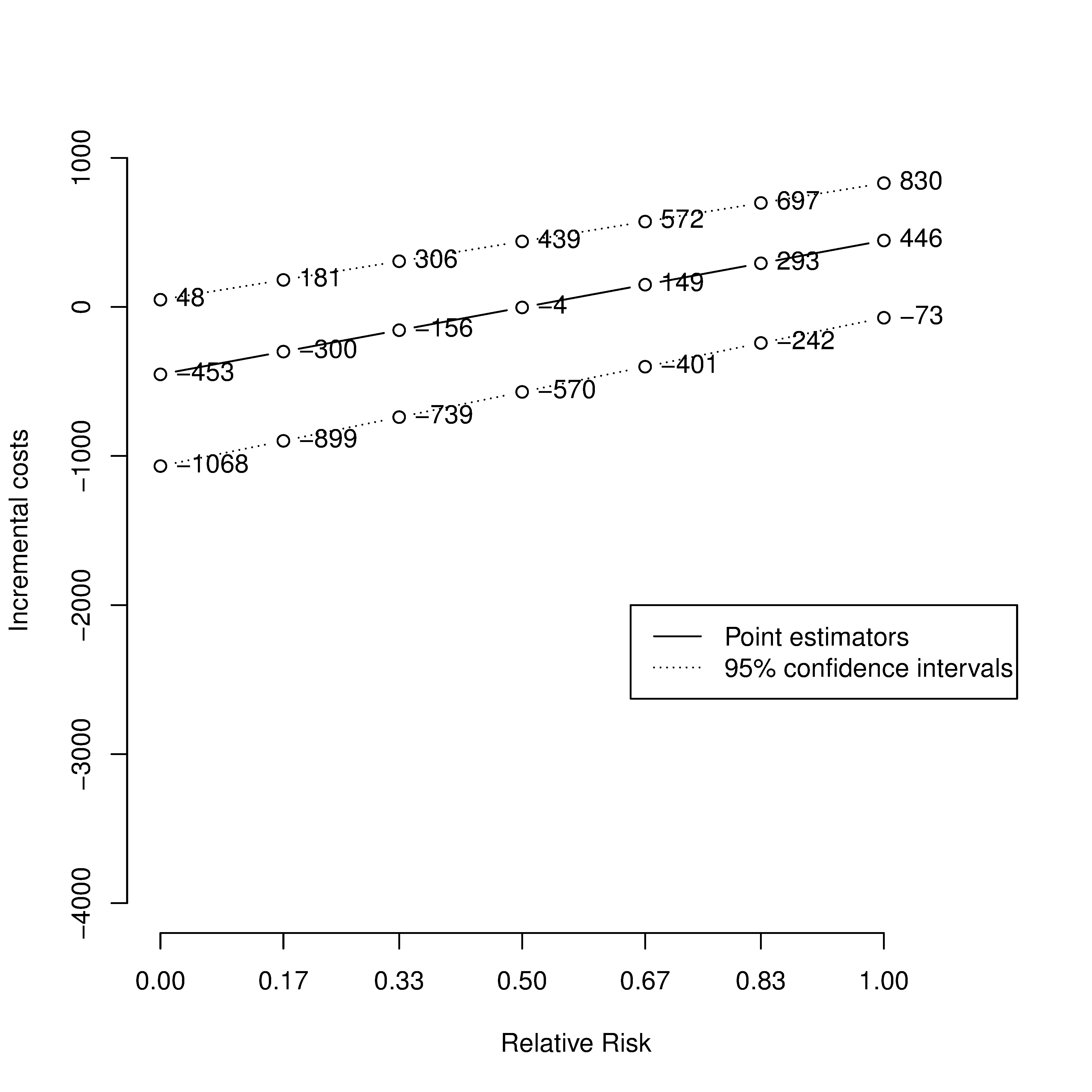
**

**Fig B. Sensitivity analysis: €2,201 for the DRG.**


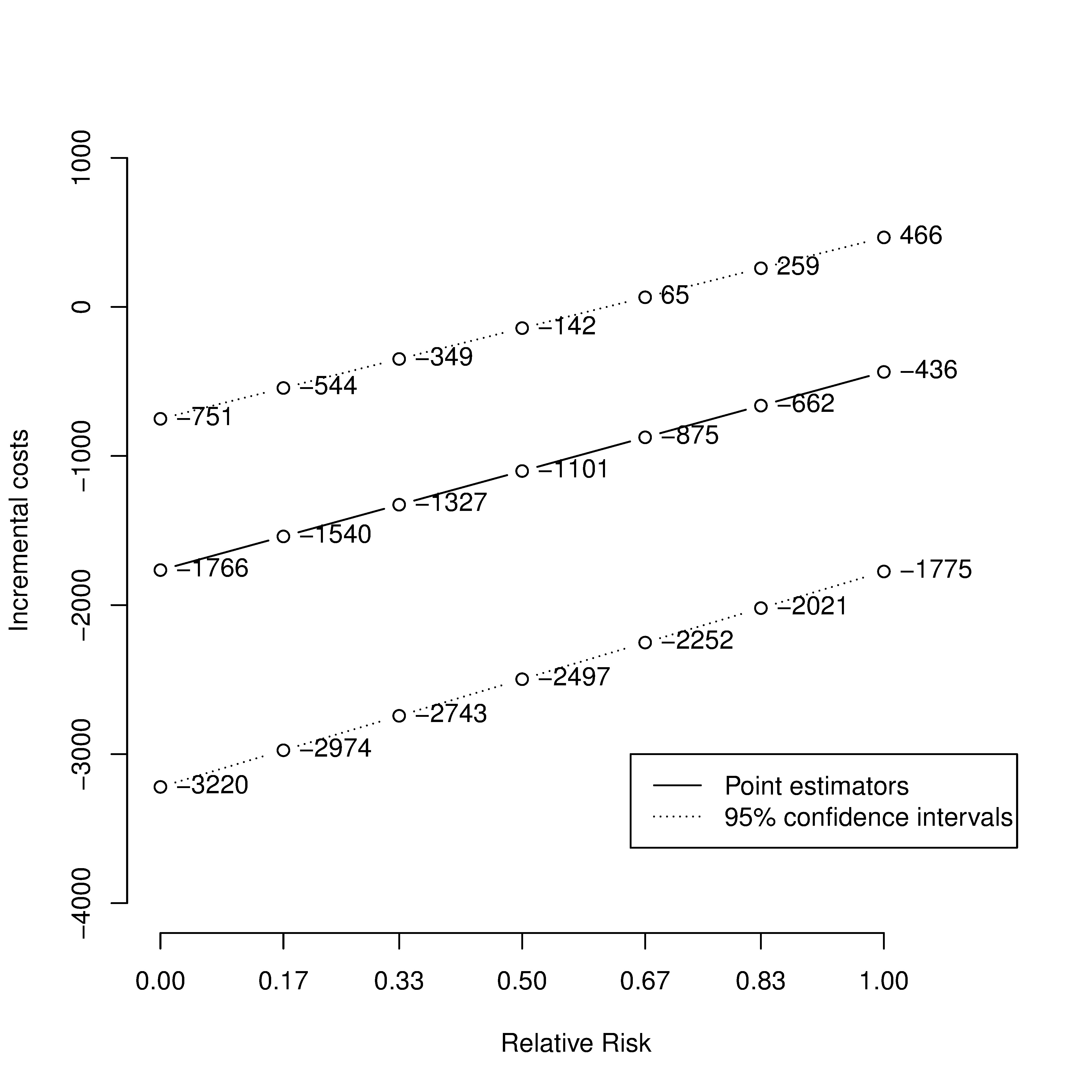


**Fig C. Sensitivity analysis: Staff time saving factor of 1 applied to the duration of FSA.**


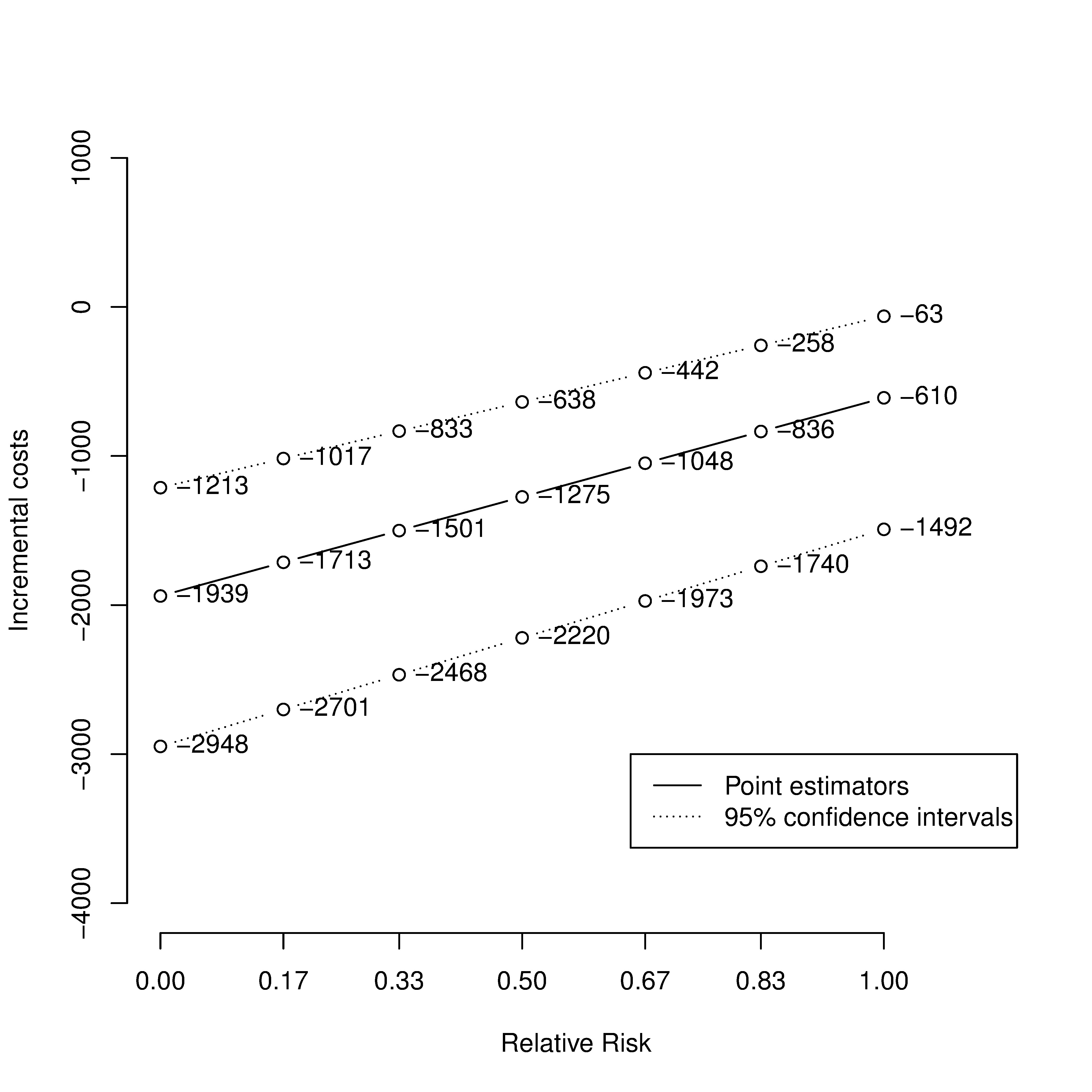


**Fig D. Sensitivity analysis: 53 minutes for the duration of FSA.**


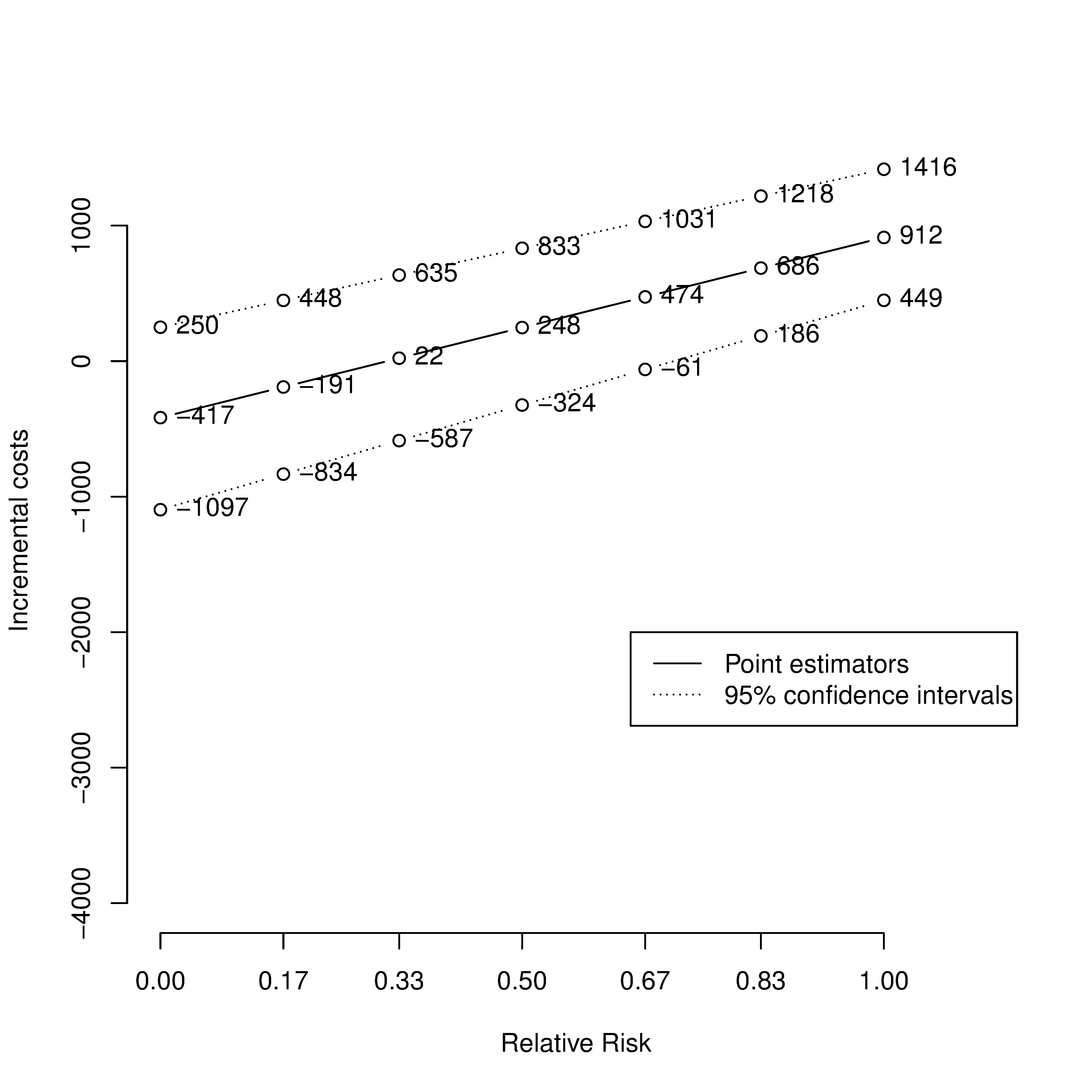


**Fig E. Sensitivity analysis: 13 minutes for the duration of FSA.**


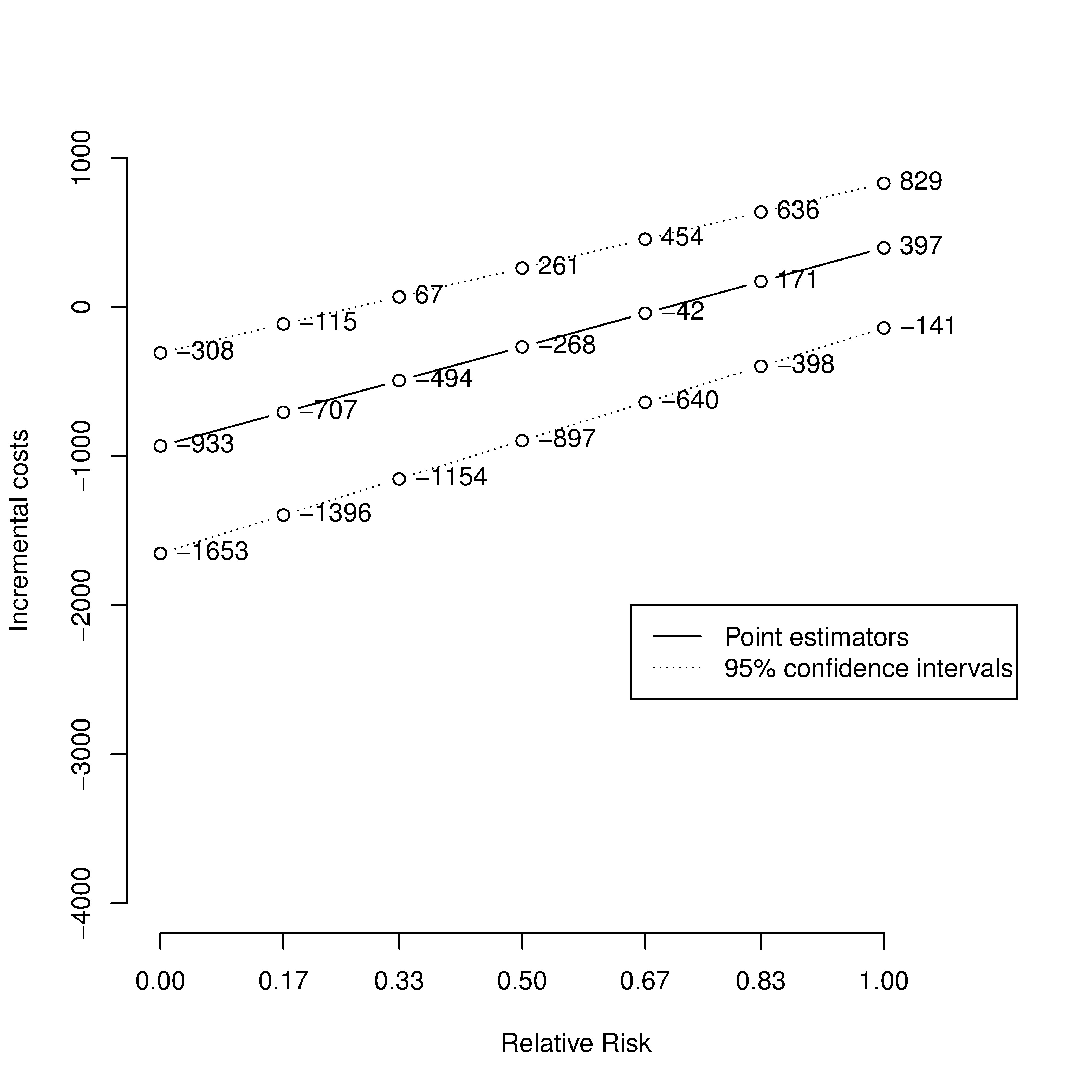


**Fig F. Sensitivity analysis: 83 minutes for the duration of ST.**


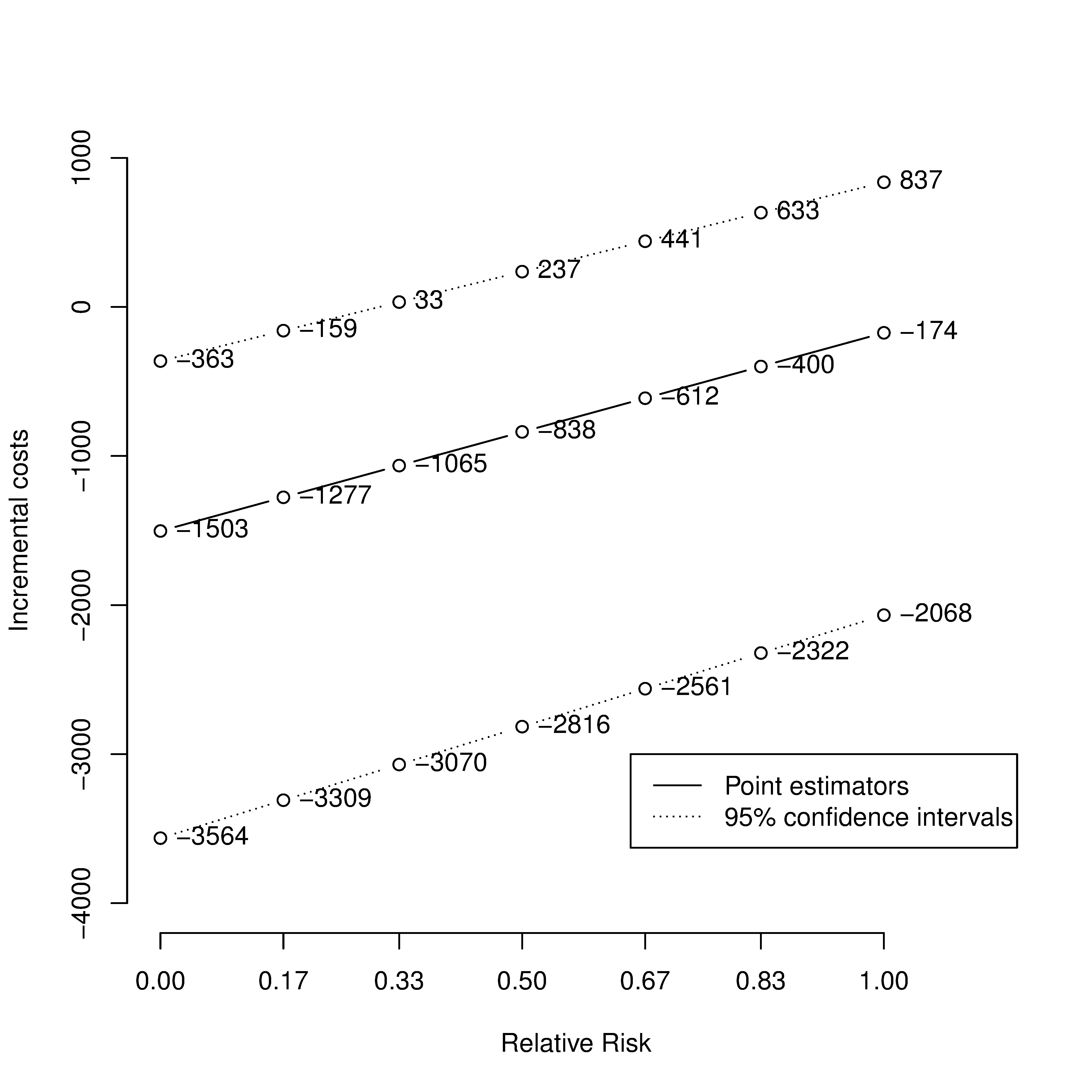


**Fig G. Sensitivity analysis: 35 minutes for the duration of ST.**

**
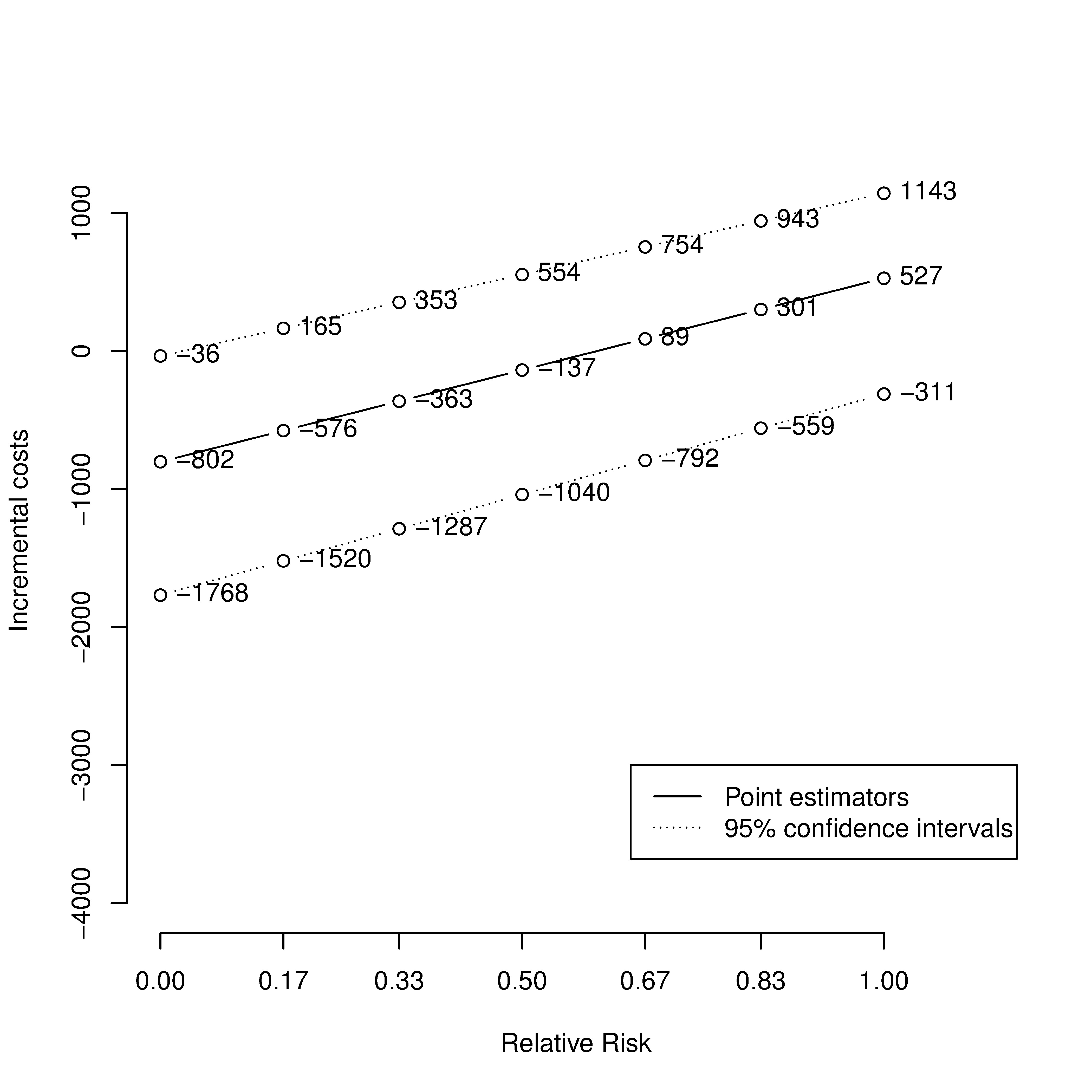
**

**Fig H. Sensitivity analysis: €800 for the costs of Bevacizumab-IRDye800CW.**

**
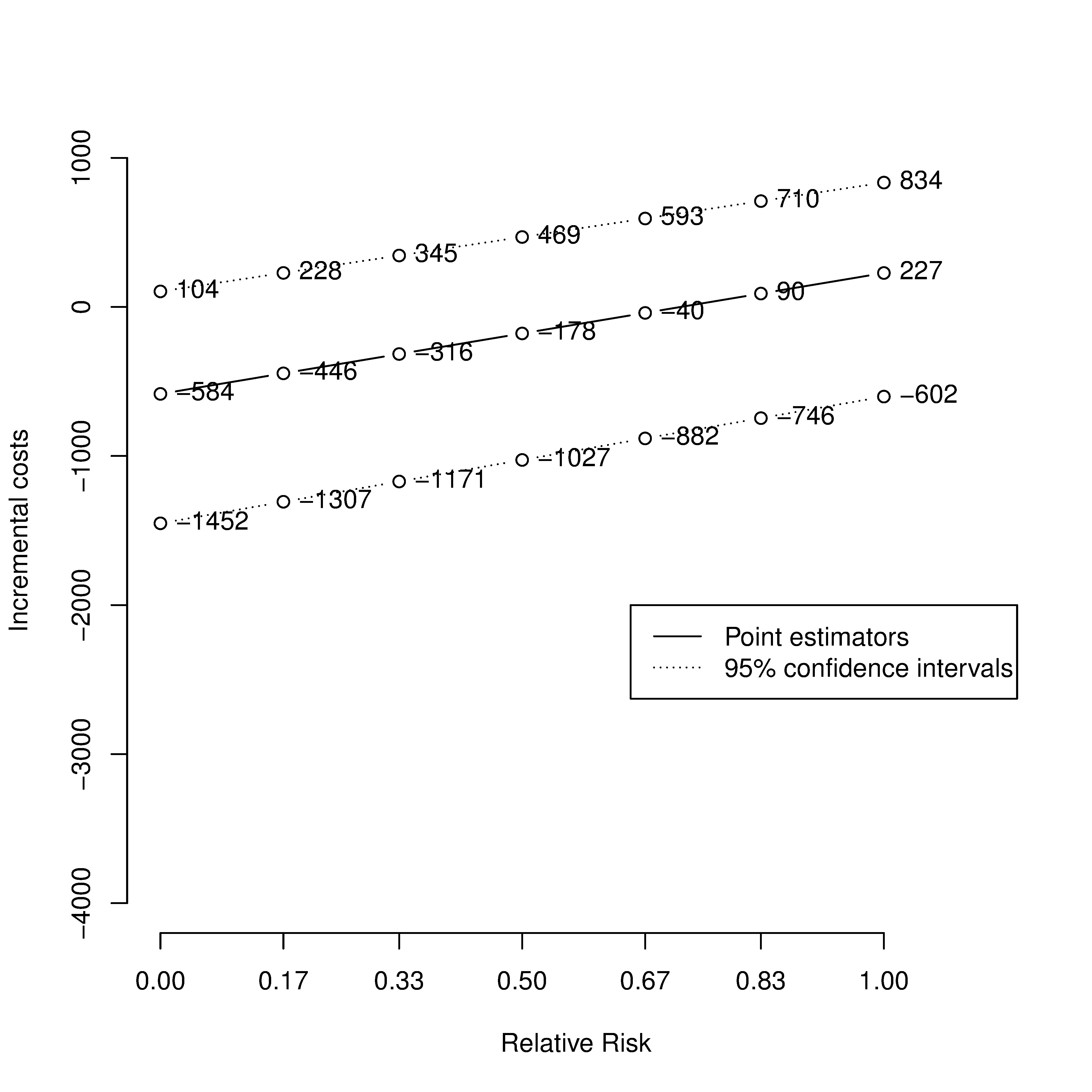
**

**Fig I. Sensitivity analysis: Proportion of positive margins regarding standard techniques of 0.18.**


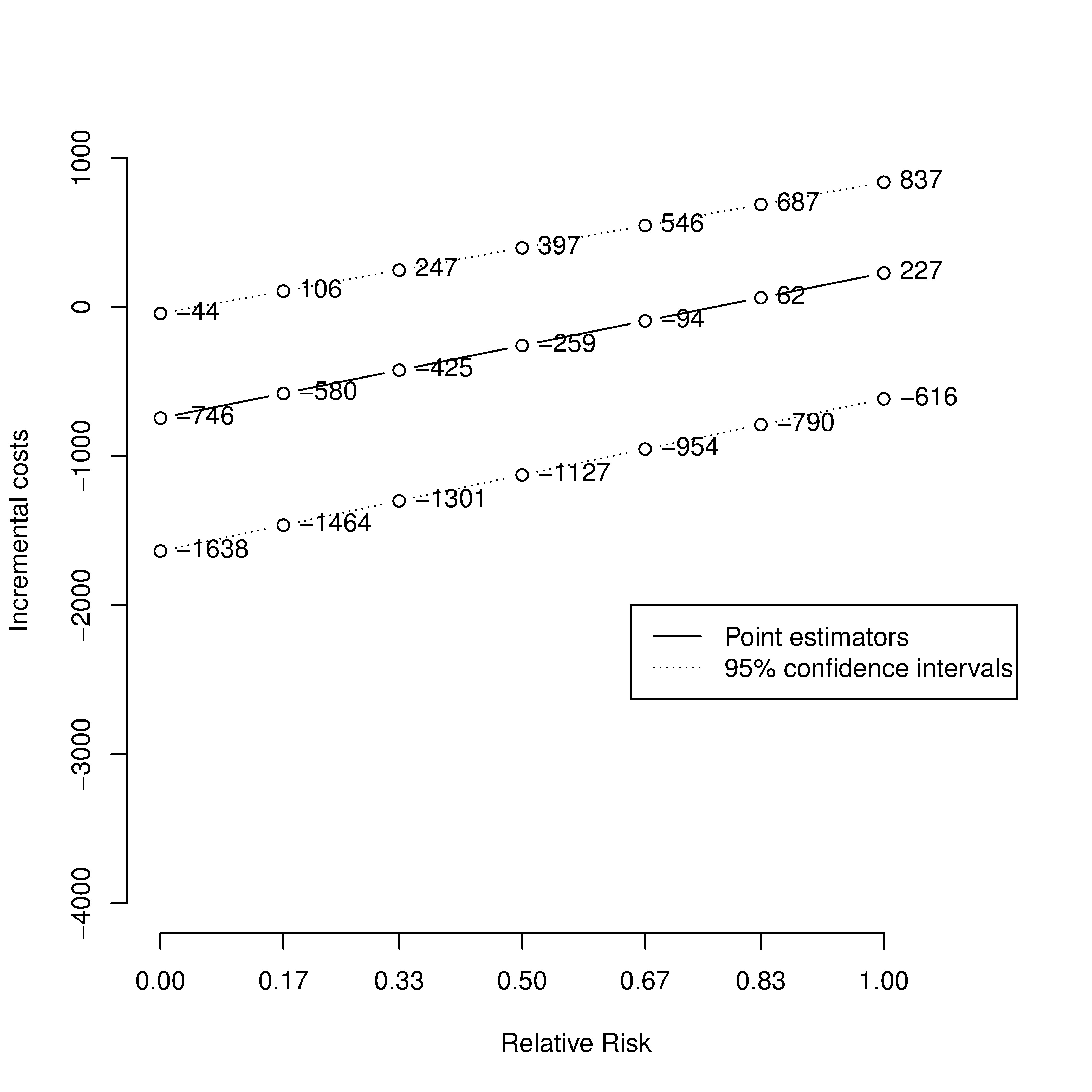


**Fig J. Structural sensitivity analysis: re-excision of negative and no re-excision of positive margins possible, exploration of incremental costs of IFMI vs. ST.**


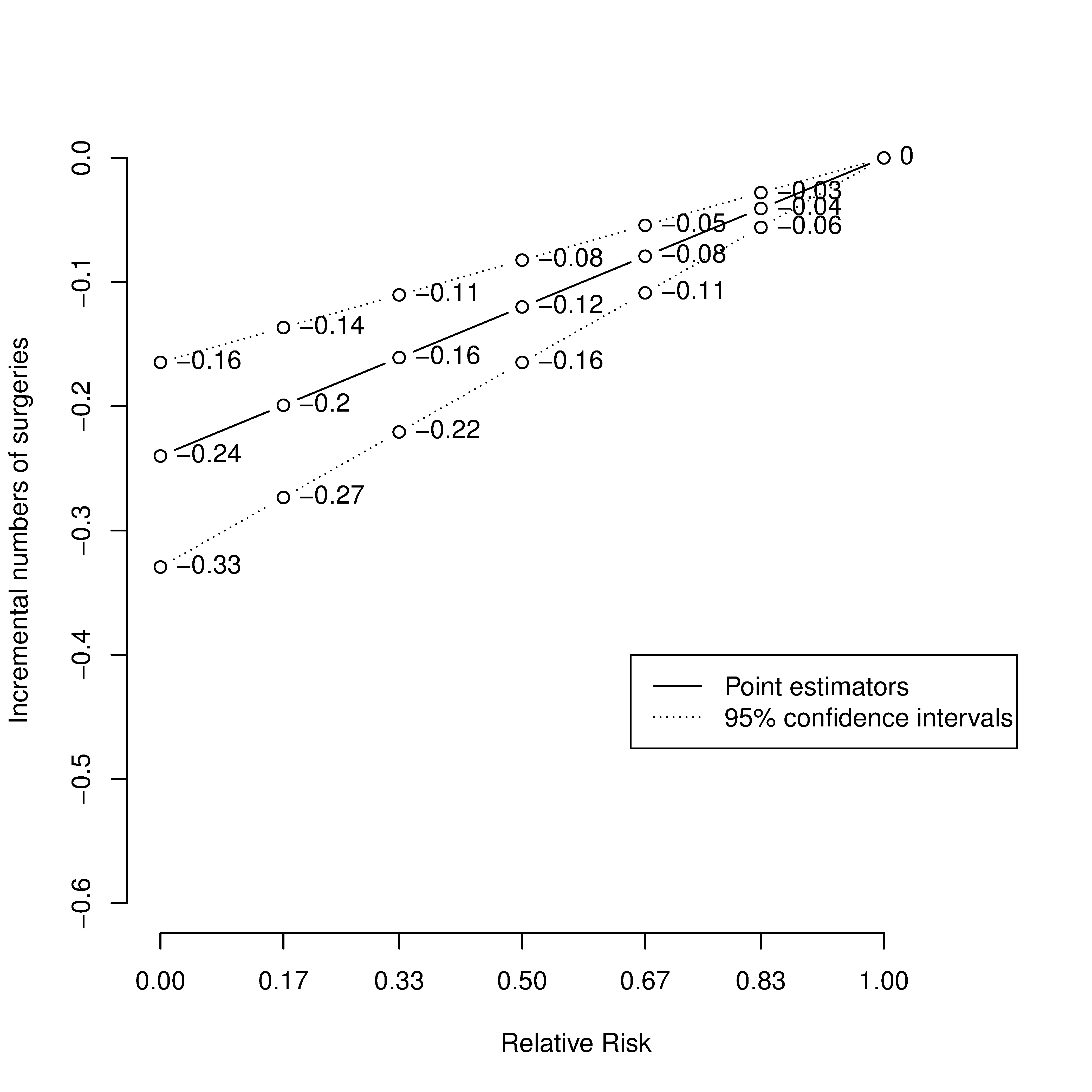


**Fig K. Structural sensitivity analysis: re-excision of negative and no re-excision of positive margins possible, exploration of incremental numbers of surgeries of IFMI vs. ST.**


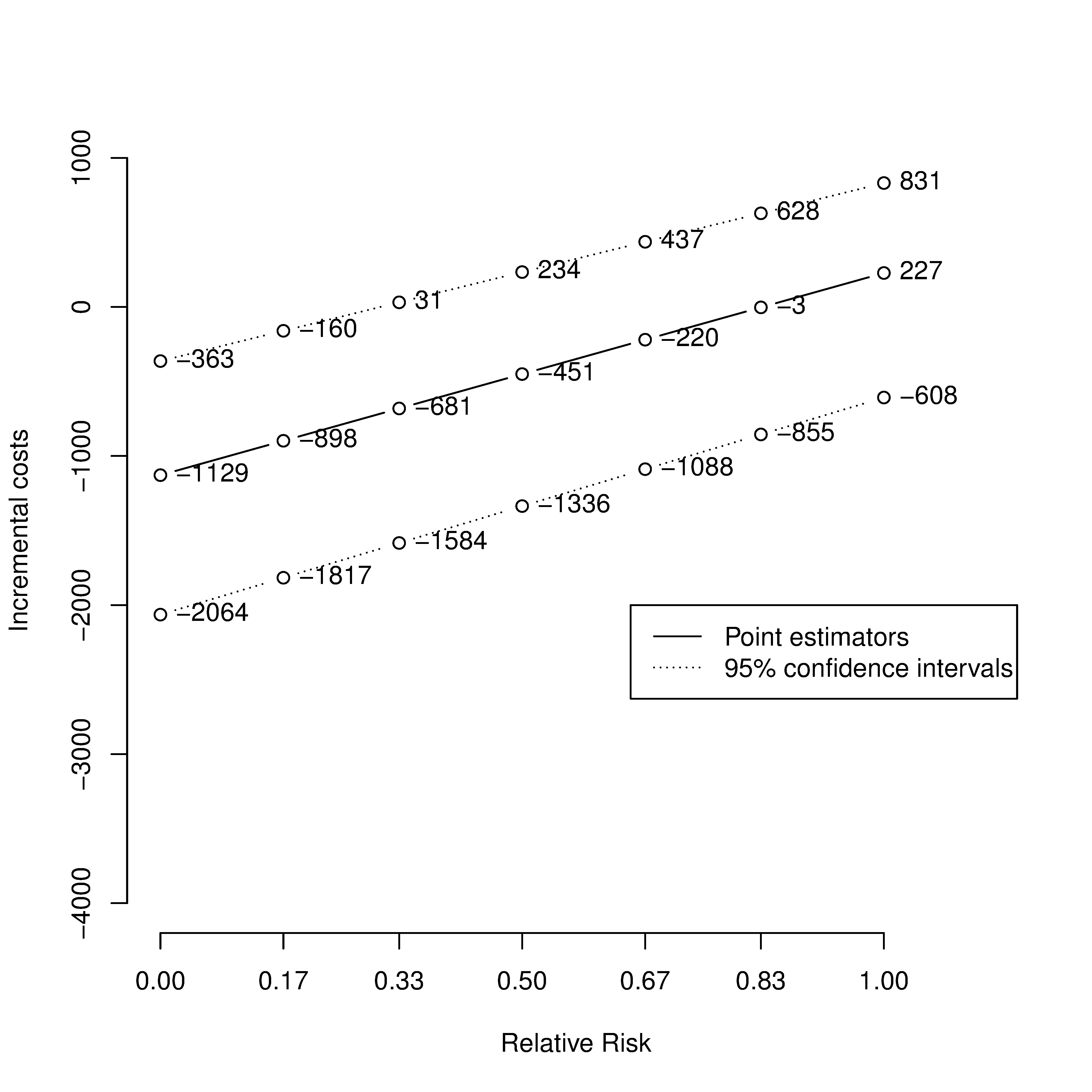


**Fig L. Structural sensitivity analysis: further surgeries independent of positive margins, exploration of incremental costs of IFMI vs. ST.**
